# Anionic polymer-coated magnetic nanocomposites for immobilization with palladium nanoparticles as catalysts for the reduction of 4-nitrophenol

**DOI:** 10.1186/s11671-023-03918-1

**Published:** 2023-11-02

**Authors:** Usana Mahanitipong, Jakkrit Tummachote, Wachirawit Thoopbucha, Wasawat Inthanusorn, Metha Rutnakornpituk

**Affiliations:** Department of Chemistry and Center of Excellence in Biomaterials, Faculty of Science, Naresuan University, Phitsanulok, 65000 Thailand

**Keywords:** Magnetite, Nanoparticle, Catalyst, Anionic polymer, Palladium

## Abstract

This study focuses on the synthesis of magnetite nanoparticles (MNP) coated with poly(poly(ethylene glycol) methacrylate) (PPEGMA) and/or poly(acrylic acid) (PAA) to anchor palladium nanoparticles (Pd) for their application as recyclable catalysts in the reduction of 4-nitrophenol (4NP). It was hypothesized that the abundance of oxygen atoms in PPEGMA enabled coordination with the Pd and provided good water dispersibility of the nanocomposites, while anionic PAA stabilized Pd and reduced the catalyst aggregation through electrostatic repulsion. Three different polymer coatings on MNP (PAA, PPEGMA, and PAA-co-PPEGMA polymers) were investigated to assess their influence on both the catalytic activity and reusability of the catalysts. Transmission electron microscopy (TEM) analysis indicated the distribution of spherical Pd nanoparticles (3–5 nm in diameter) and MNP (9–12 nm in diameter). Photocorrelation spectroscopy (PCS) revealed an average hydrodynamic size of the catalysts ranging from 540 to 875 nm in diameter, with a negative charge on their surface. The Pd content of the catalysts ranged from 4.30 to 6.33% w/w. The nanocomposites coated with PAA-co-PPEGMA polymers exhibited more favorable catalytic activity in the 4NP reduction than those coated with PAA or PPEGMA homopolymers. Interestingly, those containing PAA (e.g., PAA and PAA-co-PPEGMA polymers) exhibited good reusability for the 4NP reduction with a slight decrease in their catalytic performance after 26 cycles. This indicates the important role of carboxyl groups in PAA in maintaining high tolerance after multiple uses.

**Graphical abstract:**

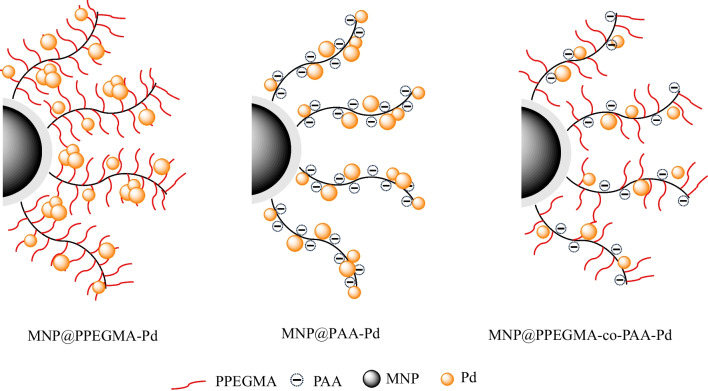

**Supplementary Information:**

The online version contains supplementary material available at 10.1186/s11671-023-03918-1.

## Introduction

In recent years, there has been significant interest in the development and synthesis of nanomaterials due to their unique properties. Among these nanomaterials, magnetite nanoparticle (MNP) has gained considerable attention in many applications, e.g., magnetic resonance imaging (MRI), separation techniques, biomedicine, biotechnology, catalysis, and more [1]. MNP exhibits excellent characteristics, such as a high surface area, small size, good stability, biocompatibility, ease of preparation, reusability, low cost, low toxicity, and convenient magnetic isolation [2–4]. However, MNP tends to self-aggregate, forming uncontrollable large clusters due to magnetic, Van der Waals, and gravitational forces. This aggregation leads to the loss of nanoscale properties and limits their applications [5]. Additionally, bare MNP is prone to oxidation and their magnetic properties often diminish or disappear in highly chemically active environments [6].

To address these challenges, the development and synthesis of polymer-coated MNP have garnered significant interest in various research fields. Polymer coatings on MNP provide protection against self-aggregation through steric repulsion, thereby improving stability and dispersibility in different media [5]. Moreover, polymers with functional groups, such as amino, oxygen, carboxylate, and more, serve as versatile platforms for immobilizing DNA, proteins, heavy metals, catalysts, and other substances [12–14]. This has led to applications in magnetic resonance imaging, controlled drug release, environmental studies, magnetic separation, hyperthermia treatment of tumor cells, and many others [7–11].

Palladium (Pd) homogeneous catalysts have gained recognition for their excellent catalytic activity and selectivity in chemical synthesis. However, these catalysts present certain limitations, such as the need for purification process post-reaction and challenges with recyclability [15]. As a result, extensive research has focused on the development of heterogeneous Pd catalysts, which can be easily separated during reactions and readily recycled. Despite their advantages, some heterogeneous Pd catalysts still exhibit lower catalytic activity and selectivity compared to homogeneous counterparts, and the issue of Pd leaching remains a challenge in chemical synthesis [16]. Recently, numerous studies have explored the synthesis of magnetite nanoparticle (MNP) coated with polymers to serve as magnetically separable supports for functional Pd catalysts [17–20]. These catalysts have shown promising catalytic activity in the reduction reaction of 4-nitrophenol (4NP), as evidenced by various reports in the literature [21–27].

Water contamination caused by organic compounds, including dyes, chlorinated solvents, fertilizers, phenols, and nitroaromatic compounds, has become a serious international concern [21]. Among these compounds, 4NP stands out as a particularly challenging pollutant due to its high biological and chemical stability, making it resistant to natural degradation [21]. 4NP is a by-product generated by various industries, including herbicides, pesticides, pharmaceuticals, and synthetic dyes [22]. Its persistence in the environment categorizes it as an environmental contaminant [23]. Of growing concern is the increasing presence of 4NP in industrial wastewater, which ultimately finds its way into rivers, thereby impairing the issue of water pollution [24]. Recognizing its detrimental impact, the US Environmental Protection Agency (US EPA) has listed 4NP as a priority pollutant [25]. The compound poses health risks to humans, affecting the central nervous system, kidneys, and liver, and can lead to the production of methemoglobin, causing anemia, as well as skin and eye irritation [4, 26]. Consequently, the removal of nitroaromatic compounds from the environment has gained significant importance, and the reduction of 4NP using transition metal catalysts presents an economically viable and safe solution [27].

**Fig. 1:**
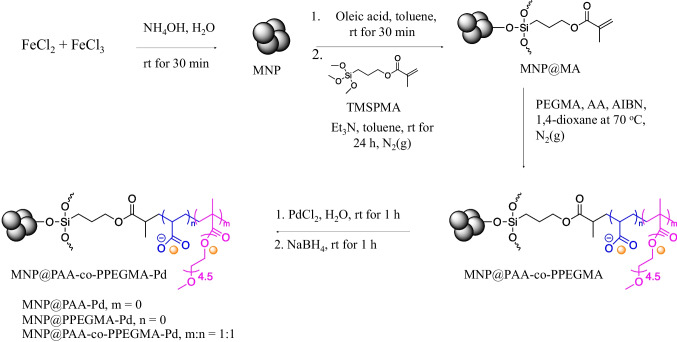
Schematic for the synthesis of MNP@polymer-Pd nanocomposite

**Fig. 2:**
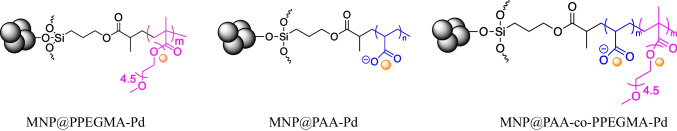
Structure of MNP@polymer-Pd nanocomposites synthesized in this work

This study presents a facile approach for the synthesis of a magnetic reusable nanocomposite, MNP@polymer-Pd, as a catalyst for the reduction of 4NP to 4-aminophenol (4AP) using NaBH_4_ as a reducing agent under mild conditions (Fig. 1). The nanocomposite consists of MNP serving as the core, which can respond to an external magnetic field. To immobilize the Pd catalyst, poly(poly(ethylene glycol) methacrylate) (PPEGMA) and/or poly(acrylic acid) (PAA) were grafted onto MNP surface. Water-soluble PPEGMA improves the dispersibility of the nanocomposite in water, and its oxygen-bearing groups enable the immobilization of the Pd catalyst. PAA, a weak polyelectrolyte, provides excellent hydrophilicity, high electronegativity, and strong coordination interactions [28]. Coating PAA on MNP helps stabilize the particles and prevent aggregation through electrostatic and steric repulsion [29–31]. The influence of different polymer coatings on MNP, specifically PAA homopolymers, PPEGMA homopolymers, and PAA-co-PPEGMA copolymers, was investigated to assess their impact on both the catalytic activity and recycling capability of the catalysts (Fig. 2).

## Experimental

### Materials

All chemicals were used without purification. Iron(II) chloride (FeCl_2_, 99+%), iron(III) chloride (FeCl_3_, 98%), poly(ethylene glycol) methacrylate (PEGMA, 300 g/mol), acrylic acid (AA, 99%), sodium borohydride (NaBH_4_, 99%, Acros), 1,4-dioxane (99.8%), and toluene (99.5%) were purchased from Acros Organics. 3-(Trimethoxy silyl)propyl methacrylate (TMSPMA, 98%), 2,2′-azobis(2-methylpropionitrile) (AIBN, 98%), palladium(II) chloride (PdCl_2,_ 99%), 4NP, ammonium hydroxide (NH_4_OH, 30% for analysis) were purchased from Sigma-Aldrich. Oleic acid (68%) and triethylamine (TEA, 97%) were purchased from Carlo Erba.

### Synthesis of methacrylate-functionalized MNP (MNP@MA)

The mixture of FeCl_3_ (1.66 g, 6.14 × 10^–3^ mol) and FeCl_2_ (1.00 g, 5.03 × 10^–3^ mol) in deionization water (20 mL) was stirred for 10 min at room temperature, followed by an addition of 25% NH_4_OH solution (20 mL), and the mixture was stirred for another 30 min to gain black precipitate of MNP. After magnetically separated, washed with water (3 × 20 mL) and then acetone (2 × 10 mL), the particle was added into the mixture of oleic acid (2 mL, 6.34 × 10^–3^ mol), TMSPMA (5.15 mL, 2.17 × 10^–2^ mol), and triethylamine (0.5 mL) in 25 mL of toluene, and it was stirred at room temperature under N_2_ atmosphere for 24 h. The synthesized MNP@MA was magnetically separated from the dispersion, repeatedly washed with hexane, 1,4-dioxane, and dried *in vacuo*.

### Synthesis of polymer-coated MNP (MNP@polymer) through a free radical polymerization

PAA-coated MNP (MNP@PAA) was herein explained as an example of the synthesis. The molar ratio of AA monomer to AIBN, a radical initiator, for the polymerization was 200:1. In a typical synthesis, acrylic acid (AA) (0.6019 mL, 17.52 × 10^–3^ mol) and AIBN (0.0144 g, 8.76 × 10^–5^ mol) were dissolved in dried 1,4-dioxane (16 mL) with magnetic stirring and then the solution was degassed for 30 min. MNP@MA (120 mg) was added and the solution was heated up to 70 °C with stirring for 6 h to obtain MNP@PAA. The particle was then magnetically separated, washed with 1,4-dioxane, then acetone, and dried *in vacuo*. MNP@PPEGMA and MNP@PAA-co-PPEGMA were synthesized via the same procedure (100:100:1 molar ratio of AA/PEGMA/AIBN in the case of copolymerization).

### Synthesis of MNP@polymer-Pd nanocomposite

MNP@polymer (100 mg) was redispersed in deionization water (3 mL) via sonication for 15 min. Then, palladium(II) chloride (16.75 mg, 9.45 × 10^–5^ mol) in deionization water (7 mL) was added dropwise, followed by an addition of 1 M HCl (20 µL). The dispersion was vigorously stirred at room temperature for 1 h. Then, NaBH_4_ (35 mg, 9.26 × 10^–4^ mol) in deionization water (3 mL) was introduced into the mixture to reduce Pd^2+^ to Pd^0^ and then the reaction was stirred for another 1 h. Finally, MNP@polymer-Pd nanocomposite was separated by a magnet, washed, and dried *in vacuo*.

### Reduction of 4NP using MNP@polymer-Pd nanocomposites as catalysts

The catalytic activity of MNP@polymer-Pd nanocomposites was evaluated via the reduction of 4NP to obtain 4AP as the product. In a typical reduction process, aqueous 4NP solution (2.00 mL, 1.25 × 10^–4^ M) and freshly prepared NaBH_4_ solution were added to quartz cuvettes with magnetic stirring at room temperature. Then, 1%, 5% or 10%mol Pd of the nanocomposites was added to the above solution. The progress of the reduction was monitored via a UV–Vis spectrophotometer at 400-nm wavelength.

### Characterization

Some of the characterization techniques employed in this study are consistent with those previously reported [12]. Fourier-transform infrared spectroscopy (FTIR, Perkin-Elmer Modle 1600 Series) in the attenuated total reflection (ATR) mode was used to characterize the functional groups of the nanocomposites. High-resolution TEM (HRTEM) was tested on FE-TEM/STEM:Thermo Scientific TALOS F200X. Field-emission scanning electron microscopy (FESEM) was performed on AperoS, Thermo Fisher Scientific with 20 kV accelerating voltage and the analysis of the elements (Fe, C, N, O) was tested via energy-dispersive X-ray (EDX) (Oxford Instruments, Oxford). The palladium content of the catalysts was evaluated using inductively coupled plasma atomic emission spectroscopy (ICP-OES, AVIO 500, Perkin-Elmer). Hydrodynamic diameter (*D*_h_) and zeta potential values of the nanocomposites were investigated via photocorrelation spectroscopy (PCS) (NanoZS4700, Malvern). Vibrating sample magnetometry (VSM) was performed on Standard 7403 Series using the magnetic fields between − 10,000 to + 10,000 G with 30-min sweep time. Thermogravimetric analysis (TGA) was tested on Thermo Plus TG8120, Rigaku, using the temperature range of 25 to 600 °C with 20 °C/min heating rate under nitrogen atmosphere. The lattice diffraction of MNP and Pd was conducted via X-ray diffraction (XRD) (Bruker D2 Phaser X-ray diffractometer).

## Results and discussion

The goal of this work was to present a facile method to prepare MNP coated with anionic polymers for use as a magnetic support for Pd immobilization having high catalytic activity; MNP was simply fabricated via a coprecipitation of Fe(III) and Fe(II) salts in NH_4_OH solution (TEM images shown in Fig. S1 in Additional file 1). PPEGMA and/or PAA were grafted on MNP via a free radical polymerization of MNP@MA. PEGMA, a hydrophilic polymer containing abundant oxygen atoms, served as coordinating sites with Pd, enhanced water dispersibility, and essentially diminished the particle self-aggregation [12]. PAA, bearing a lot of carboxylate groups, provided an additional platform for the immobilization of Pd. The main aim of this work was to study the effect of PPEGMA and PAA coating on the Pd immobilization efficiency, the catalytic activity, and the reusability for the 4NP reduction of the nanocomposites. Hence, MNPs coated with three different types of polymers: PAA homopolymers, PPEGMA homopolymers, and PAA-co-PPEGMA copolymers, were synthesized and used in these studies.

### Characterization of MNP@polymer-Pd nanocomposites

**Fig. 3:**
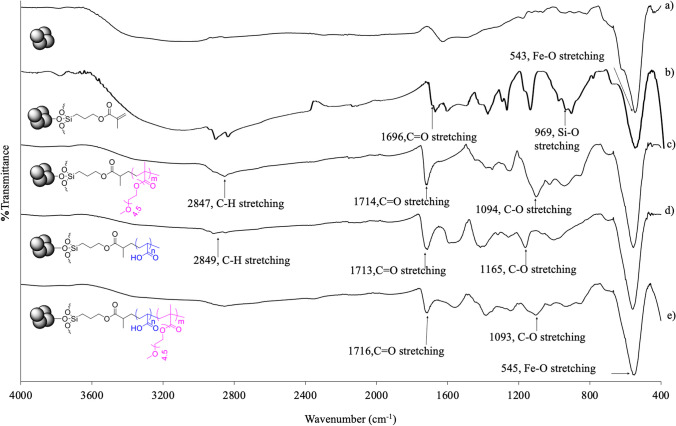
FTIR spectra of **a** bare MNP, **b** MNP@MA, **c** MNP@PPEGMA, **d** MNP@PAA, and **e** MNP@PPEGMA-co-PAA

The functional groups of bare MNP, MNP@MA, MNP@PPEGMA, MNP@PAA, and MNP@PPEGMA-co-PAA were characterized via FTIR spectrophotometry (Fig. 3). The results indicated that all of nanocomposites containing MNP clearly showed the characteristic signal of Fe–O stretching vibration at 543 cm^−1^. Figure 3b exhibits the characteristic signals of MNP@MA at 971 cm^−1^ (Si–O stretching), 1589 cm^−1^ (C=C stretching) and 1712 cm^−1^ (C=O stretching). After coating with the polymers, the strong and sharp signals of C=O stretching and those of C–O stretching at 1065–1094 cm^−1^ appeared at 1713–1716 cm^−1^, indicating the successful modification of MNP surface with the polymers (Fig. 3c–e).

**Fig. 4:**
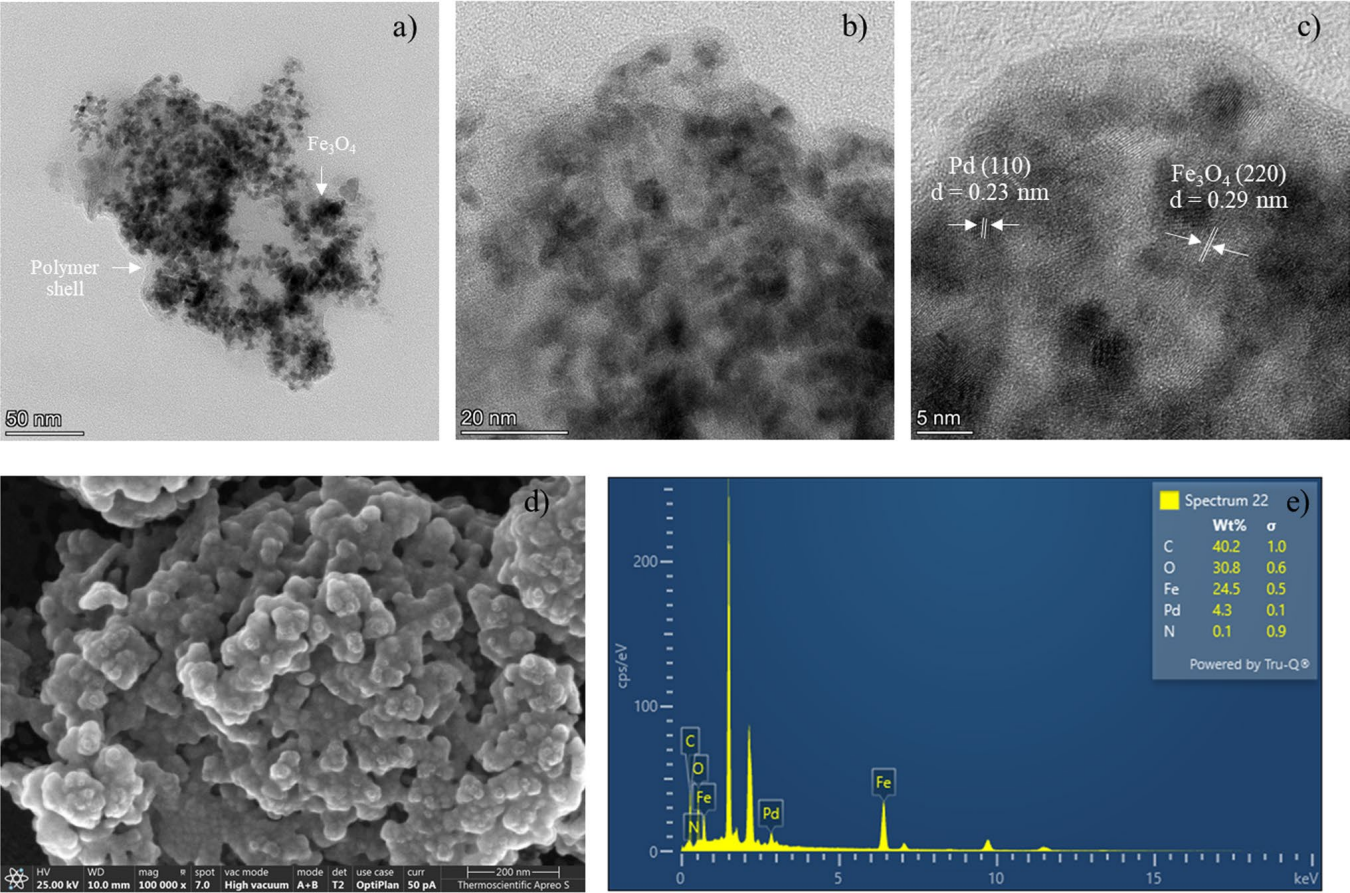
**a**–**c** TEM images, **d** an SEM image, and **e** an EDX pattern of MNP@PAA-Pd

The TEM images shown in Fig. 4a–c provide information about the particle size, distribution, the polymer layer, and the lattices of Pd and MNP in MNP@PAA-Pd. HRTEM analysis confirmed the presence of spherical Pd nanoparticles (3–5 nm in diameter) and MNP nanoparticles (9–12 nm in diameter) embedded within the polymer layers. Notably, the (110) planes (*d* = 0.23 nm) of Pd and the (220) planes (*d* = 0.29 nm) of MNP were clearly observed in Fig. 4c. An FESEM image displayed a certain level of nanoclustering, with the spherical particles coated by polymer shells (Fig. 4d). Furthermore, EDX revealed the presence of the palladium signal, signifying the successful immobilization of Pd on the surface of the nanocomposites (Fig. 4e). The TEM, SEM and EDX of MNP@PPEGMA-Pd, and MNP@PPEGMA-co-PAA-Pd exhibit the same results as those of MNP@PAA-Pd and are shown in supplementary information (Additional file 1: Fig. S2 and Fig. S3). XRD technique was utilized to analyze the phase crystallinity of these three nanocomposites. The results revealed characteristic diffraction peaks at specific angles (crystal planes of MNP): 30.2° (220), 35.5° (311), 43.2° (400), 53.6° (422), 57.2° (511), and 62.9° (440) (JCPDS 89-3854) [16]. Additionally, the peaks were observed at 40.1°, 46.7°, and 68.1°, aligning with the (111), (200), and (220) crystal planes of Pd (JCPDS 46-1043). The XRD patterns shown in Additional file 1: Fig. S4 indicate that there is no significant difference in the crystal structure of these three nanocomposites (Additional file 1: Fig. S4).

**Fig. 5:**
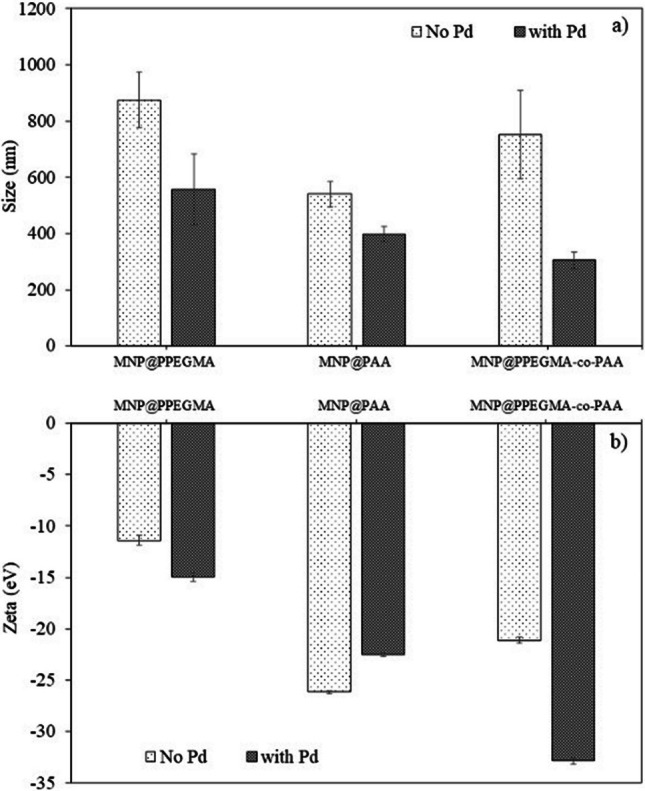
**a** Particle size distributions and **b** zeta potential values of MNP@PPEGMA, MNP@PAA, and MNP@PPEGMA-co-PAA with and without Pd

*D*_h_ and zeta potential values of MNP@PPEGMA, MNP@PAA, and MNP@PPEGMA-co-PAA before and after Pd loadings in water were analyzed using PCS (Fig. 5). The results showed that their average *D*_h_ was in the range of 540–875 nm in diameter. MNP@PAA exhibited smaller average *D*_h_ compared to those of MNP@PPEGMA and MNP@PPEGMA-co-PAA and this was attributed to the relatively higher polarity of MNP@PAA, resulting in a more uniform dispersion compared to the other two. When considering the pKa of PAA (pKa = 4.5) [32], the formation of carboxylate anions on the MNP surface can occur in the pH 7 solution. The anionic nature of the carboxylate anions contributed to electrostatic repulsion with other particles, and this can essentially minimize the formation of MNP nano-aggregation. Interestingly, *D*_h_ of the particles loaded with Pd has decreased significantly compared to those without Pd (Fig. 5a), and this was attributed to the net reduction in the polarity of the nanocomposite owing to the Pd-polymer interaction. The zeta potential values for MNP@PPEGMA, MNP@PAA, and MNP@PPEGMA-co-PAA were found to be − 11.4, − 26.2, and − 21.1 eV, respectively (Fig. 5b). This indicated that MNP@PAA exhibited the highest degree of negative charge due to the presence of abundant carboxylate groups compared to MNP@PPEGMA and MNP@PPEGMA-co-PAA.

**Fig. 6:**
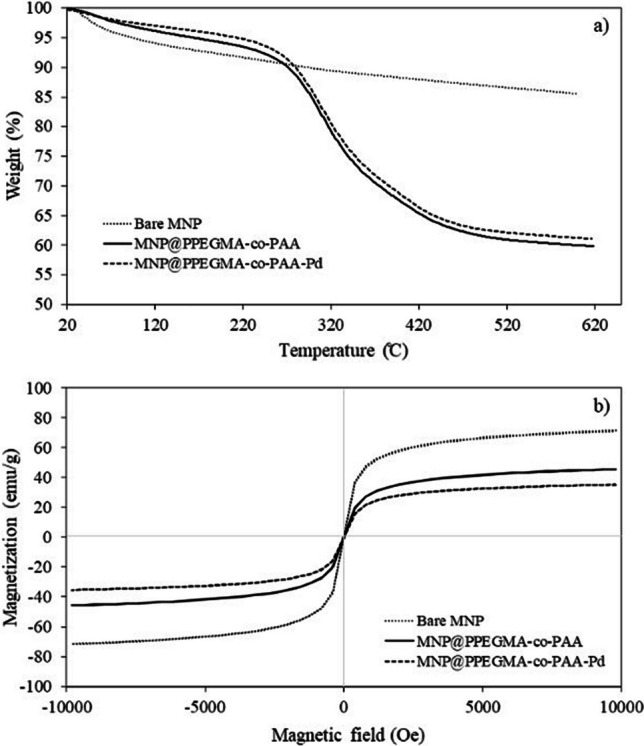
**a** TGA thermograms and **b** magnetization curves of bare MNP, MNP@PPEGMA-co-PAA, and MNP@PPEGMA-co-PAA-Pd with and without Pd

The organic and inorganic compositions of bare MNP, MNP@PPEGMA-co-PAA, and MNP@PPEGMA-co-PAA-Pd were evaluated through TGA (Fig. 6a). The TGA curves exhibited a slight weight loss of approximately 2.8–5.2% at 100 °C, which was ascribed to the evaporation of surface-bound water and volatile compounds. Notably, the TGA profiles of MNP@PPEGMA-co-PAA and MNP@PPEGMA-co-PAA-Pd exhibited a substantial weight reduction of about 38.4–39.7% within the temperature range of 200–550 °C. This notable weight loss was attributed to the presence of organic polymers. These findings provide conclusive evidence for the presence of a polymer layer on the surface of these nanocomposites.

The magnetic properties of bare MNP, MNP@PPEGMA-co-PAA, and MNP@PPEGMA-co-PAA-Pd were investigated using VSM (Fig. 6b). The magnetization-field (M-H) curves of these samples revealed their superparamagnetic behavior. Saturation magnetization (*M*_s_) values were determined to be 72 emu/g for bare MNP, 46 emu/g for MNP@PPEGMA-co-PAA, and 35 emu/g for MNP@PPEGMA-co-PAA-Pd. It appears that the coating of PPEGMA-co-PAA and the immobilization of Pd on the MNP surface contribute to a lower M_s_ value compared to bare MNP. In good agreement with the weight loss results from TGA, this reduction in *M*_s_ can be attributed to the increased existence of organic materials in the nanocomposites. Despite the decrease in magnetic properties, all the nanocomposites exhibited a good response to a permanent magnet. The *M*_s_ values provide a means to quantify the composition of individual components (MNP, PPEGMA-co-PAA, and Pd) within the nanocomposites. Prior to Pd loading, the nanocomposites comprised 36% of PPEGMA-co-PAA and 64% of MNP. These percentages were recalculated after the introduction of Pd, maintaining the same MNP to PPEGMA-co-PAA ratio. The analysis revealed that the nanocomposites consisted of 15% Pd, 54% MNP, and 31% PPEGMA-co-PAA.

**Fig. 7:**
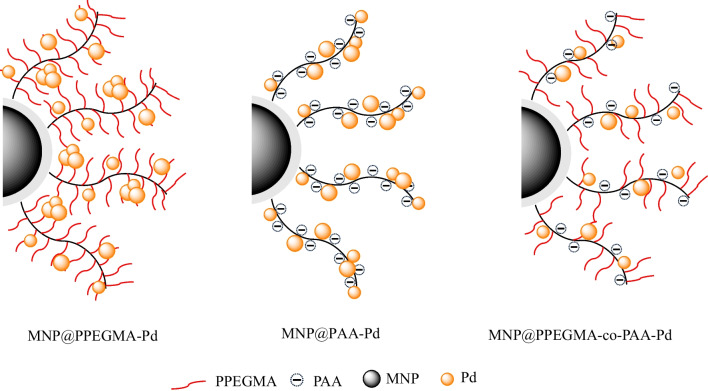
The illustration showing the proposed nano-aggregation of Pd in each polymer structure

The Pd contents of MNP@PPEGMA-Pd, MNP@PAA-Pd, and MNP@PPEGMA-co-PAA-Pd catalysts were determined using ICP-OES. The Pd contents of the catalysts ranged from 4.3 to 6.3% w/w (Table 1). Interestingly, MNP@PPEGMA-Pd exhibited the highest Pd content compared to MNP@PPEGMA-Pd and MNP@PPEGMA-co-PAA-Pd. This was attributed to some nano-aggregation of Pd on the brush-like structure of PPEGMA. On the other hand, MNP@PPEGMA-co-PAA-Pd showed the lowest Pd content in this series and this was probably due to the existence of the spacing between PAA and PPEGMA repeating units of the statistical copolymers, which somewhat diminished the Pd aggregation on the polymer. This rationalization agreed well with the catalytic activity results discussed in the later section. The illustration showing the proposed nano-aggregation of Pd in each polymer structure is shown in Fig. 7.

**Table 1:** Pd contents of MNP@PPEGMA-Pd, MNP@PAA-Pd, and MNP@PPEGMA-co-PAA-Pd

Catalyst	Pd content (%w/w)	Pd loading (mmol/g)	Pd incorporation (%)
MNP@PPEGMA-Pd	6.33	59.49	63.31
MNP@PAA-Pd	5.80	54.53	58.03
MNP@PPEGMA-co-PAA-Pd	4.30	40.38	42.97

### Catalytic activity of synthesized catalysts

To assess the catalytic activity of the catalysts, the reaction of 4NP with NaBH_4_ reducing agent in water at room temperature to form 4AP was employed as a model reaction. Initially, the aqueous solution of 4NP exhibited a maximum UV absorbance peak at 300 nm. Upon addition of NaBH_4_, the absorbance peak shifted to 400 nm, indicating the formation of 4NP anion. Subsequently, upon introduction of the synthesized catalysts into the solution containing 4NP anion and NaBH_4_, the UV absorbance of 4NP anion at 400 nm decreased rapidly due to its conversion to 4AP. A new absorbance peak at 330 nm corresponding to 4AP emerged. Complete disappearance of the absorbance peak of 4NP anion indicated the completion of the 4NP reduction process. The catalytic reduction of 4NP using MNP@PPEGMA-Pd, MNP@PAA-Pd, and MNP@PPEGMA-co-PAA-Pd catalysts was monitored at 400 nm using a UV–Vis spectrometer over time. The catalytic activity of the synthesized catalysts for the 4NP reduction was quantified through pseudo-first-order kinetics, with the catalytic activity reported as the rate constant (*k*) using Eq. (1) [12]:

1 $${\text{Ln}}(A_{{\text{t}}} /A_{0} ) = - kt$$

where *A*_t_ and *A*_0_ were absorbance of 4NP anion at selected reaction and initial times, and *k* value was the reaction rate constant obtained from the slope of the linear plot of Ln (*A*_t_/*A*_0_) versus *t*.

Turnover frequency (TOF) of the catalysts can be calculated from Eq. (2) [33]:

2 $${\text{TOF}} = {\text{(mol of converted substate/mol of Pd)}}/t$$

**Fig. 8:**
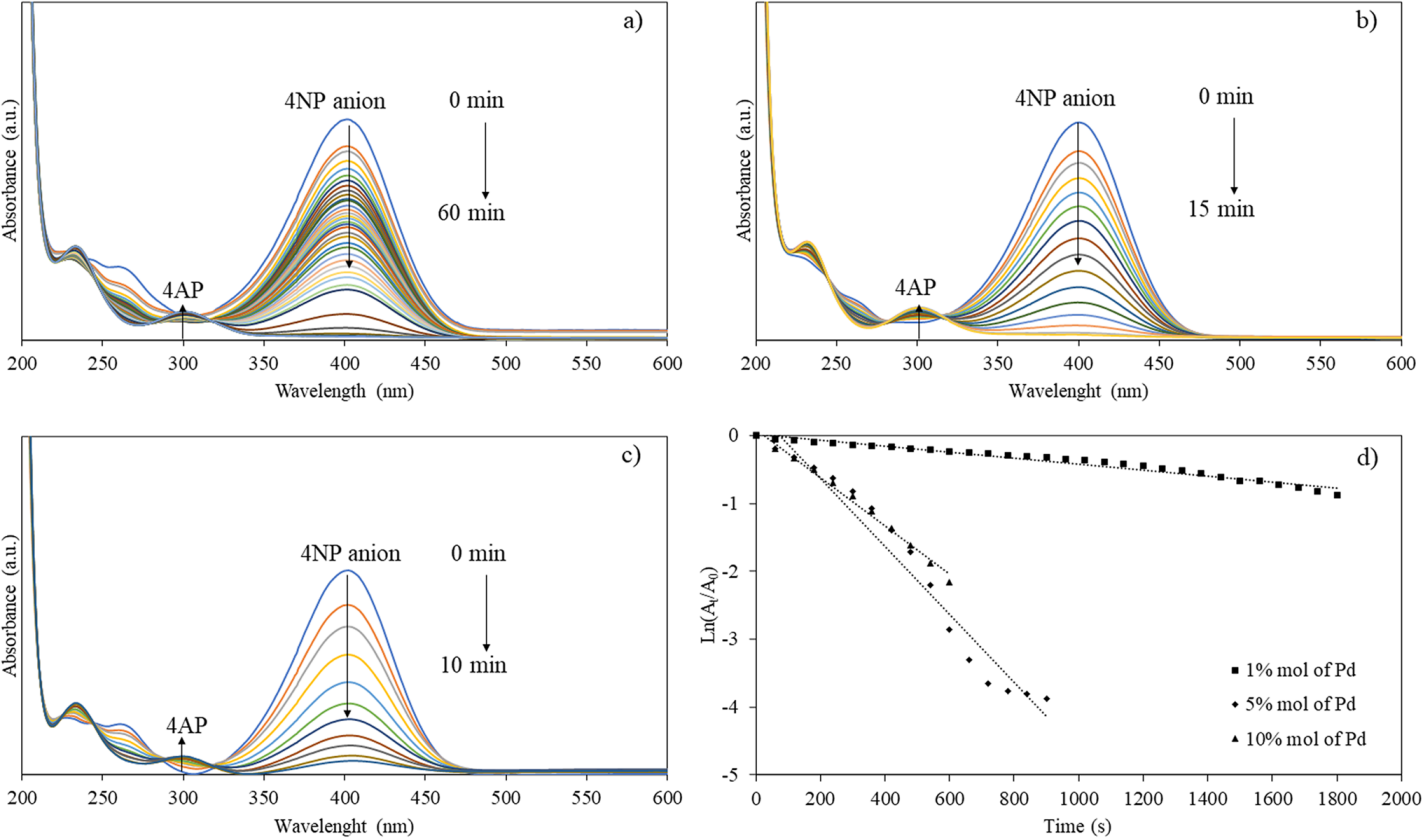
UV–Vis absorption spectra of the 4NP reduction using MNP@PPEGMA-Pd catalyst with **a** 1% mol of Pd, **b** 5% mol of Pd, **c** 10% mol of Pd, and **d** the plot of Ln (*A*_t_/*A*_0_) versus the reaction time using 1, 5, and 10% mol of Pd

To evaluate the catalytic activity of synthesized catalysts, the amounts of Pd of the nanocomposites were varied from 1, 5 to 10% mol of Pd. The catalytic efficiency (*k* value) of the catalysts was determined using pseudo-first-order reaction kinetics as described in Eq. (1). The results revealed promising catalytic activity for the reduction of 4NP as the examples of UV–Vis absorption spectra when using MNP@PPEGMA-Pd catalyst (Fig. 8a–c). Notably, the catalysts with 10% mol of Pd exhibited the highest catalytic activity, with *k* values ranging from 0.0009 to 0.0059 s^−1^ and TOF ranging from 26.02 to 57.60 h^−1^ (Fig. 8d and Table 2). In contrast, the catalysts with 1% mol of Pd showed *k* values ranging from 4 × 10^–5^ to 0.0002 s^−1^, while those with 5% mol of Pd exhibited *k* values ranging from 0.0005 to 0.0025 s^−1^. Evidently, an increase in the percentage of Pd resulted in higher catalytic activity. The catalytic activity for the reduction of 4NP using MNP@PAA-Pd and MNP@PPEGMA-co-PAA-Pd revealed similar results to those of MNP@PPEGMA-Pd. The first-order plot and the %conversion plot of all samples are shown in supplementary information (Additional file 1: Fig. S5).

**Table 2:** The catalytic activity of the catalysts for the 4NP reduction using various % mol Pd

Catalyst name	% mol of Pd	Time (min)	*k* value (s^−1^)	%Conversion	TOF (h^−1^)
MNP@PPEGMA-Pd	1	60	0.0003	97.26	97.26
	5	15	0.0031	97.92	78.34
	10	*10*	0.0044	88.56	53.14
MNP@PAA-Pd	1	60	4 × 10^–5^	56.92	56.92
	5	15	0.0005	79.51	63.60
	10	10	0.0009	43.37	26.02
MNP@PPEGMA-co-PAA-Pd	1	60	0.0002	97.31	97.31
	5	15	0.0025	95.74	76.59
	10	10	0.0059	96.00	57.60

Furthermore, when focusing on the homopolymer-coated catalysts using 10% mol of Pd, it was observed that MNP@PPEGMA-Pd exhibited better catalytic activity (*k* value = 0.0044 s^−1^, TOF = 53.14 h^−1^) when compared to MNP@PAA-Pd (*k* value = 0.0009 s^−1^, TOF = 26.02 h^−1^). Interestingly, the Pd-immobilized MNPs coated with the copolymer (PPEGMA-co-PAA) demonstrated even further improved catalytic activity (*k* value = 0.0059 s^−1^, TOF = 57.60 h^−1^) compared to the PAA- and PPEGMA-coated particles. This enhancement can be attributed to the interplay between the negative charge of PAA and the non-charge in PPEGMA, which created a favorable environment for Pd immobilization and minimized Pd nano-aggregation. This rationalization aligns well with the lower Pd content observed in MNP@PPEGMA-co-PAA-Pd when compared to the other two catalysts.

### Reusability of the catalysts for the 4NP reduction

**Fig. 9:**
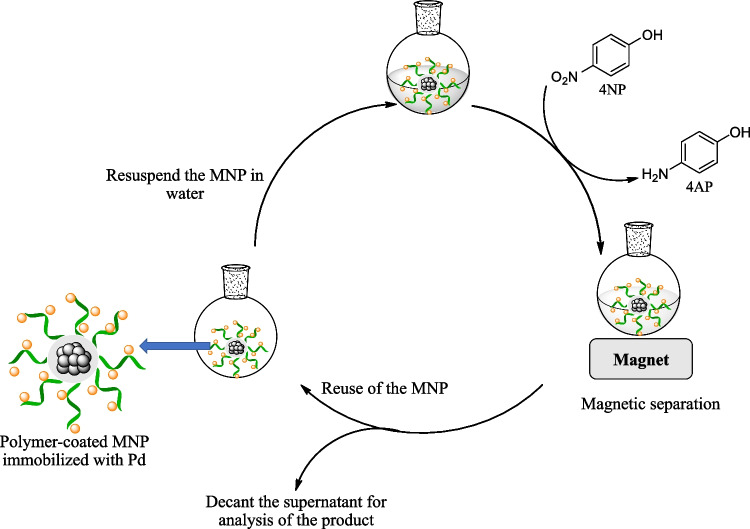
The process for reusability testing of the synthesized catalysts for the reduction of 4NP

**Fig. 10:**
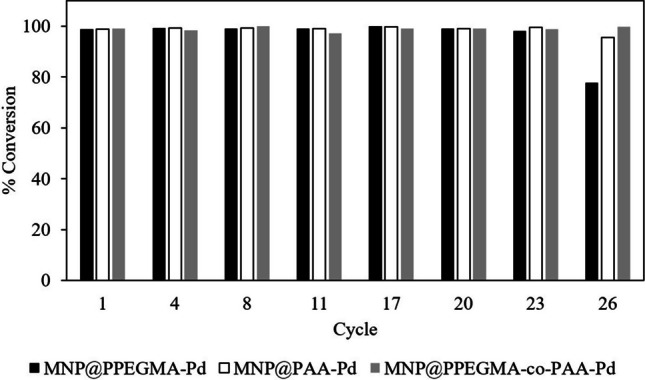
Reusability of MNP@PPEGMA-Pd, MNP@PAA-Pd, and MNP@PPEGMA-co-PAA-Pd catalysts

The reusability of catalysts is a crucial factor for its environmental applications; the synthesized catalysts were evaluated for their performance in the reduction of 4NP using 10 mol% of Pd catalyst as the model. The process for reusability testing of the catalysts is shown in Fig. 9. The results demonstrated that the synthesized catalysts exhibited favorable catalytic reusability for the 4NP reduction, maintaining their activity even after multiple cycles. Remarkably, they could be reused for at least 26 cycles with only a slight decrease in catalytic activity (Fig. 10). In the initial cycle, they achieved a high conversion of 99%. Even after 23 cycles, the catalytic activity remained robust, with conversions of 98%, 99%, and 98% for MNP@PPEGMA-Pd, MNP@PAA-Pd, and MNP@PPEGMA-co-PAA-Pd catalysts, respectively. However, at the 26th cycle, the catalytic activity of the PPEGMA-based catalyst sharply declined to 78% conversion, while the catalysts with PAA exhibited sustained high catalytic activity with conversions of 95% (MNP@PAA-Pd) and 99% (MNP@PPEGMA-co-PAA-Pd), respectively. These results indicate that the carboxylic groups present in PAA enhance the catalytic activity of the catalysts during the reaction. Moreover, the analysis of Pd leaching from the catalysts revealed the exceptionally low Pd leaching in the catalytic reduction reactions (total over 26 runs < 1.5 ppm) or below 0.26 ppm per cycle, further supporting their stability and suitability for repeated use (Table S1 in Supplementary information).

The catalytic performance of MNP@PPEGMA-Pd, MNP@PAA-Pd, and MNP@PEGMA-Co-PAA-Pd in the reduction of 4NP was benchmarked against several catalysts previously reported in the literature. A comparative analysis, including the type of metals, reaction time, conversion rate, and rate constant (*k*), is presented in Table 3. The data underlines the efficiency of various metal-based catalysts in the 4NP reduction process. Notably, the results indicate that these three nanocomposites exhibit high *k* values, which are comparable to both the catalysts documented in other studies and those from our prior research.

**Table 3:** The comparison of the amount of catalyst, time, conversion, and *k* for the 4-nitrophenol reduction of the nanocomposites synthesized in this work with other catalysts reported in studies

Catalysts	Type of metals	Time of reaction (min)	Conversion (%)	*k* (s^−1^)	References
AuNPs/TiO_2_	Au	6	Nearly 100	0.0105	[27]
CuO	Cu	16	98.7	0.0017	[34]
Tetraaniline/Ag	Ag	20	97	0.0025	[35]
Cellulose-PAA/PVA-Cu	Cu	14	99.87	0.0055	[36]
Cellulose-PAA/PVA-Ni	Ni	14	98.38	0.0012	[36]
AgNCs@CF-g-PAA	Ag	6	96	0.0086	[37]
Fe_3_O_4_@CMC-Pd	Pd	15	93.50	0.0031	[38]
Fe_3_O_4_@CMC/PDEAEMA-Pd	Pd	20	96.12	0.0027	[38]
MNP@PPEGMA-Pd	Pd	*10*	88.56	0.0044	This work
MNP@PAA-Pd	Pd	10	43.37	0.0009	This work
MNP@PEGMA-co-PAA-Pd	Pd	10	96.00	0.0059	This work

NPs, nano particles, PVA, poly(vinyl alcohol); NCs, nanoclusters; CF, cotton fiber; CMC, carboxymethyl chitosan; PDEAEMA, poly(2-(diethylamino)ethyl methacrylate)

## Conclusions

Magnetic nanosorbents coated with PPEGMA and/or PAA were successfully synthesized for the immobilization of Pd catalysts, enabling their application as recoverable catalysts with an assistance of a magnet for the reduction of 4NP. The incorporation of PPEGMA and/or PAA as the polymer layer in the catalysts was specifically designed to enhance their water dispersibility and catalytic activity. Remarkably, these catalysts exhibited excellent performance in the reduction of 4NP in aqueous environments and demonstrated the ability to be easily recycled for a minimum of 26 cycles, achieving a conversion rate of at least 78%. This notable reusability highlights the potential of these catalysts for sustainable applications, as they maintained high catalytic activity even after multiple recycling processes. Consequently, these catalysts hold significant promise as reusable catalysts within the field of sustainability, offering a practical solution for diverse applications.

### Supplementary Information

**Additional file 1. Supplementary Information. Fig. S1** TEM images of uncoated MNP. **Fig. S2** a-c) TEM images, d) an SEM image and e) an EDX pattern of MNP@PEGMA-Pd. **Fig. S3** a-b) TEM images, c) an SEM image and d) an EDX pattern of MNP@PPEGMA-co-PAA. **Fig. S4** XRD patterns of a) MNP@PPEGMA-Pd, b) MNP@PAA-Pd, and c) MNP@PPEGMA-co-PAA-Pd catalysts. The (hkl) numbers in red represent the lattice planes of Pd and those in black are of MNP. **Fig. S5** The first-order plot and the %conversion plot of the 4NP reduction using (a) MNP@PPEGMA-Pd, (b) MNP@PAA-Pd and (c) MNP@PPEGMA-co-PAA-Pd catalysts having 1%, 5% and 10 % mol of Pd. Table S1 The Pd leaching test results of MNP@PPEGMA-Pd, MNP@PAA-Pd and MNP@PEGMA-Co-PAA-Pd when repeatedly used in 4NP reduction.

## Data Availability

The authors declare that the data supporting the findings of this study are available within the paper and its Supplementary information. Should any raw data files be needed in another format they are available from the corresponding author upon request.
