# Supplementary material for: Anionic polymer-coated magnetic nanocomposites for immobilization with palladium nanoparticles as catalysts for the reduction of 4-nitrophenol
Source: Discov Nano. 2023 Nov 2;18(1):138. doi: 10.1186/s11671-023-03918-1 (PMC10622386; doi:10.1186/s11671-023-03918-1)
Supplement: Supplementary file 1 — Additional file 1. Supplementary Information. Fig. S1 TEM images of uncoated MNP. Fig. S2 a-c) TEM images, d) an SEM image and e) an EDX pattern of MNP@PEGMA-Pd. Fig. S3 a-b) TEM images, c) an SEM image and d) an EDX pattern of MNP@PPEGMA-co-PAA. Fig. S4 XRD patterns of a) MNP@PPEGMA-Pd, b) MNP@PAA-Pd, and c) MNP@PPEGMA-co-PAA-Pd catalysts. The (hkl) numbers in red represent the lattice planes of Pd and those in black are of MNP. Fig. S5 The first-order plot and the %conversion plot of the 4NP reduction using (a) MNP@PPEGMA-Pd, (b) MNP@PAA-Pd and (c) MNP@PPEGMA-co-PAA-Pd catalysts having 1%, 5% and 10 % mol of Pd. Table S1 The Pd leaching test results of MNP@PPEGMA-Pd, MNP@PAA-Pd and MNP@PEGMA-Co-PAA-Pd when repeatedly used in 4NP reduction. [file 11671_2023_3918_MOESM1_ESM.docx]

**Anionic polymer-coated magnetic nanocomposites for immobilization with palladium nanoparticles as catalysts for the reduction of 4-nitrophenol**

**Usana Mahanitipong, Jakkrit Tummachote, Wachirawit Thoopbucha, Wasawat Inthanusorn and Metha Rutnakornpituk***

Department of Chemistry and Center of Excellence in Biomaterials, Faculty of Science, Naresuan University, Phitsanulok 65000, Thailand, Phone: +66-5596-3464, Fax: +66-5596-3401, *E-mail: [methar@nu.ac.th](mailto:methar@nu.ac.th)

**Supplementary Information (SI)**

**Fig. S1** TEM images of uncoated MNP


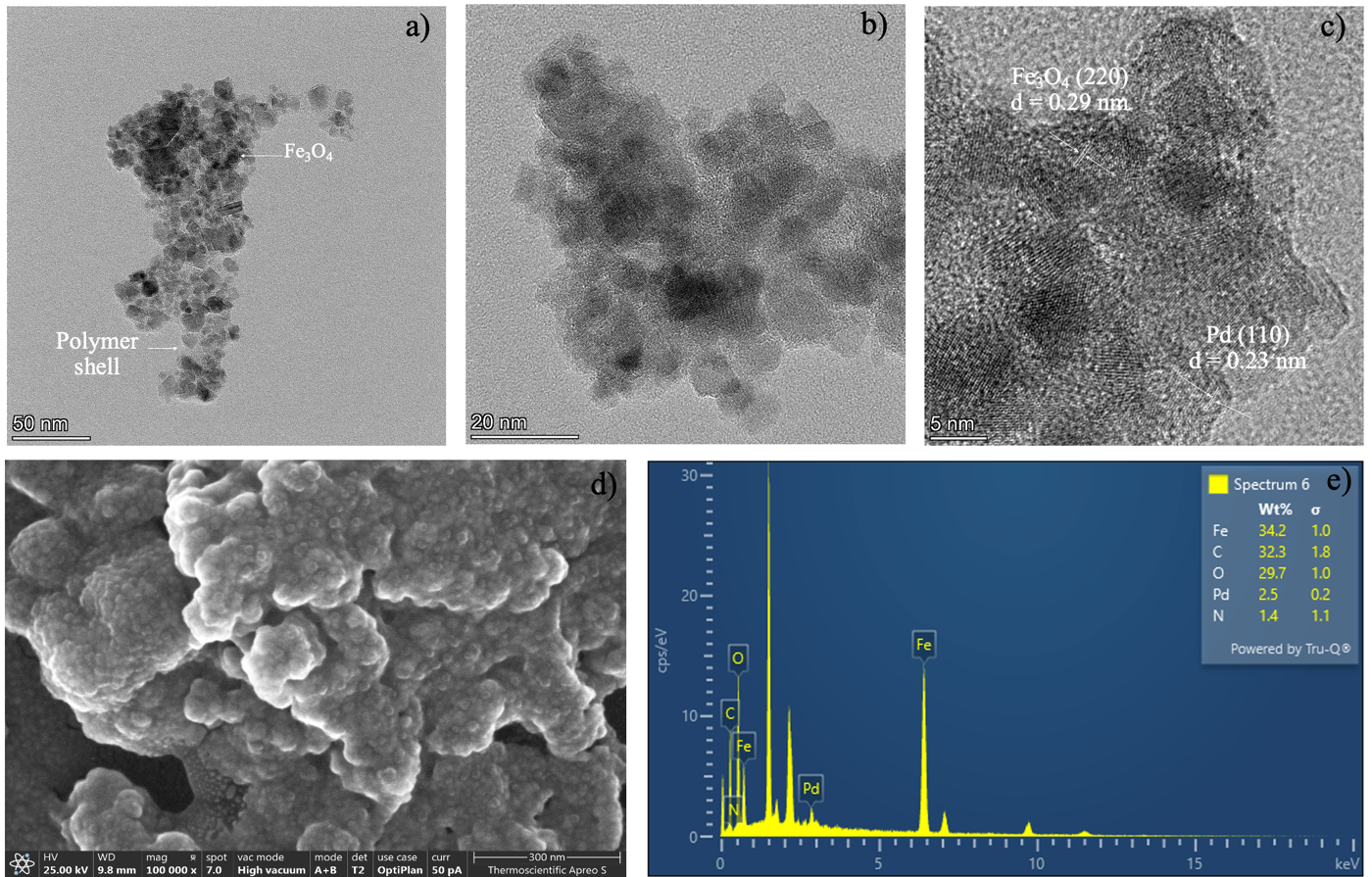


**Fig. S2** a-c) TEM images, d) an SEM image and e) an EDX pattern of MNP@PEGMA-Pd


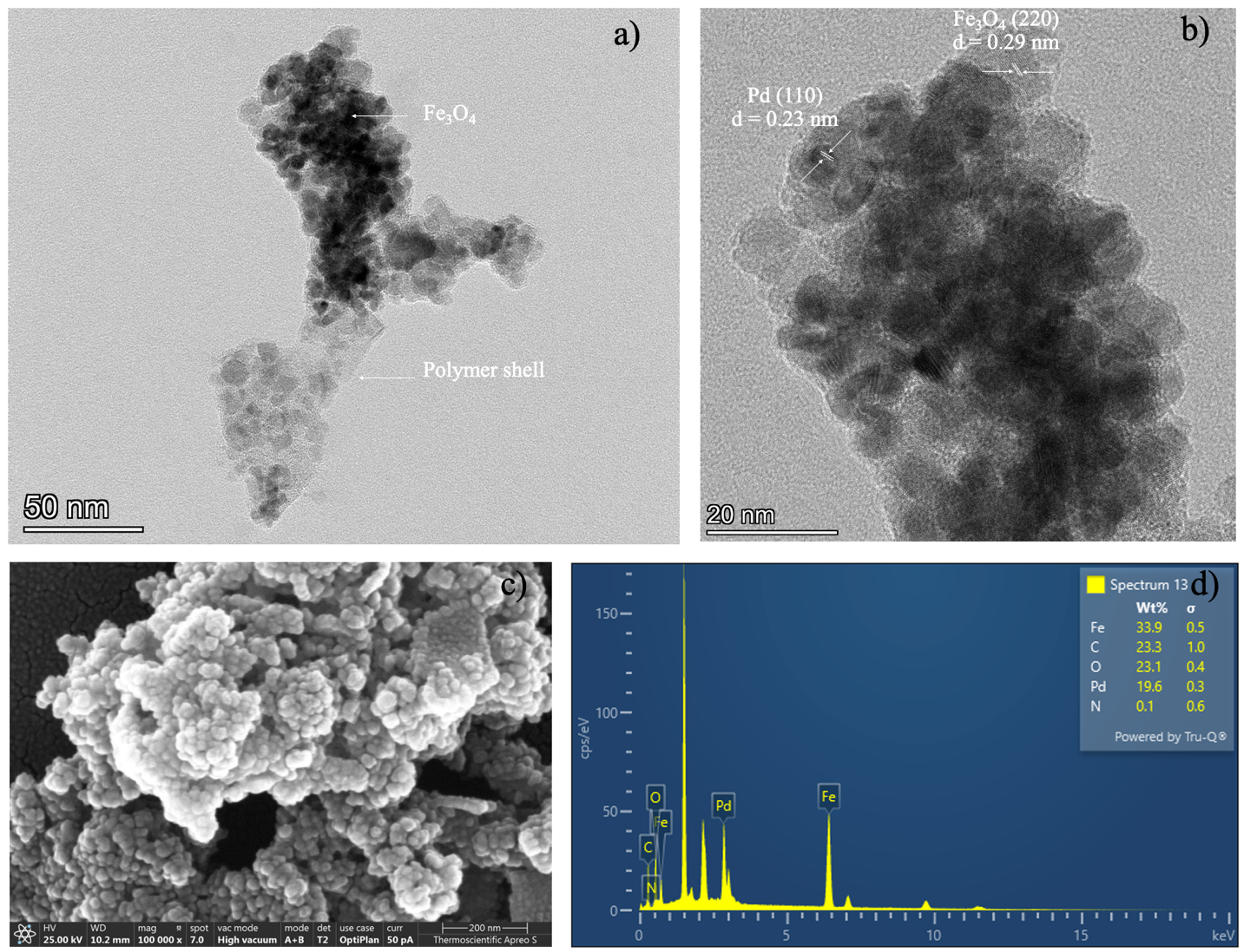


**Fig. S3** a-b) TEM images, c) an SEM image and d) an EDX pattern of MNP@PPEGMA-co-PAA

**Fig. S4** XRD patterns of a) MNP@PPEGMA-Pd, b) MNP@PAA-Pd, and c) MNP@PPEGMA-co-PAA-Pd catalysts. The (hkl) numbers in red represent the lattice planes of Pd and those in black are of MNP.

**Fig. S5** The first-order plot and the %conversion plot of the 4NP reduction using (a) MNP@PPEGMA-Pd, (b) MNP@PAA-Pd and (c) MNP@PPEGMA-co-PAA-Pd catalysts having 1%, 5% and 10 % mol of Pd

**Table S1** The Pd leaching test results of MNP@PPEGMA-Pd, MNP@PAA-Pd and MNP@PEGMA-Co-PAA-Pd when repeatedly used in 4NP reduction

| Cycle | Pd leaching (ppm) | | |
| --- | --- | --- | --- |
|  | MNP@PPEGMA-Pd | MNP@PAA-Pd | MNP@PEGMA-Co-PAA-Pd |
| 1 | 0.0733 ± 0.0079 | 0.0788 ± 0.0140 | 0.2570 ± 0.0079 |
| 2 | 0.0476 ± 0.0079 | 0.0623 ± 0.0082 | 0.0780 ± 0.0096 |
| 3 | 0.0484 ± 0.0028 | 0.0850 ± 0.0090 | 0.1329 ± 0.0034 |
| 4 | 0.1452 ± 0.0106 | 0.1285 ± 0.0101 | 0.1630 ± 0.0205 |
| 5 | 0.1782 ± 0.0031 | 0.0791 ± 0.0164 | 0.0938 ± 0.0095 |
| 8 | 0.0471 ± 0.0090 | 0.1310 ± 0.0258 | 0.0514 ± 0.0121 |
| 11 | 0.0131 ± 0.0038 | 0.0646 ± 0.0054 | 0.0669 ± 0.0015 |
| 14 | 0.0119 ± 0.0023 | 0.0912 ± 0.0030 | 0.1565 ± 0.0155 |
| 17 | 0.0277 ± 0.0030 | 0.0006 ± 0.0002 | 0.1030 ± 0.0025 |
| 20 | 0.0556 ± 0.0153 | 0.0216 ± 0.0112 | 0.0795 ± 0.0153 |
| 23 | 0.0230 ± 0.0039 | 0.0264 ± 0.0049 | 0.1099 ± 0.0082 |
| 26 | 0.0269 ± 0.0065 | 0.0035 ± 0.0018 | 0.0543 ± 0.0254 |
| Total | 0.7189 | 0.8107 | 1.4092 |
